# Is social support pre-treatment associated with prognosis for adults with depression in primary care?

**DOI:** 10.1111/acps.13285

**Published:** 2021-02-16

**Authors:** Joshua E. J. Buckman, Rob Saunders, Ciaran O’Driscoll, Zachary D. Cohen, Joshua Stott, Gareth Ambler, Simon Gilbody, Steven D. Hollon, Tony Kendrick, Edward Watkins, Nicola Wiles, David Kessler, Nomsa Chari, Ian R. White, Glyn Lewis, Stephen Pilling

**Affiliations:** 1 Centre for Outcomes Research and Effectiveness (CORE), Research Department of Clinical, Educational & Health Psychology, University College London, London, UK; 2 iCope – Camden & Islington Psychological Therapies Services – Camden & Islington NHS Foundation Trust, London, UK; 3 Department of Psychiatry, University of California Los Angeles, Los Angeles, CA, USA; 4 Statistical Science, University College London, London, UK; 5 Department of Health Sciences, University of York, York, UK; 6 Department of Psychology, Vanderbilt University, Nashville, TN, USA; 7 Primary Care, Population Sciences and Medical Education, Faculty of Medicine, University of Southampton, Southampton, UK; 8 Department of Psychology, University of Exeter, Exeter, UK; 9 Centre for Academic Mental Health, Population Health Sciences, Bristol Medical School, University of Bristol, Bristol, UK; 10 Centre for Academic Primary Care, Department of Population Health Science, Bristol Medical School, University of Bristol, Bristol, UK; 11 Division of Psychiatry, University College London, London, UK; 12 MRC Clinical Trials Unit at UCL, London, UK; 13 Camden & Islington NHS Foundation Trust, St Pancras Hospital, London, UK

**Keywords:** depression, social support, prognosis, treatment outcome, individual patient data meta-analysis

## Abstract

**Objective:**

Depressed patients rate social support as important for prognosis, but evidence for a prognostic effect is lacking. We aimed to test the association between social support and prognosis independent of treatment type, and the severity of depression, and other clinical features indicating a more severe illness.

**Methods:**

Individual patient data were collated from all six eligible RCTs (n = 2858) of adults seeking treatment for depression in primary care. Participants were rand-omized to any treatment and completed the same baseline assessment of social support and clinical severity factors. Two-stage random effects meta-analyses were conducted.

**Results:**

Social support was associated with prognosis independent of randomized treatment but effects were smaller when adjusting for depressive symptoms and durations of depression and anxiety, history of antidepressant treatment, and comorbid panic disorder: percentage decrease in depressive symptoms at 3–t months per z-score increase in social support = −4.14(95%CI:−6.91 to −1.29). Those with a severe lack Acta of social support had considerably worse prognoses than those with no lack of social support: increase in depressive symptoms at 3”4 months = 14.64%(4.25% to 26.06%).

**Conclusions:**

Overall, large differences in social support pre-treatment were associ-ated with differences in prognostic outcomes. Adding the Social Support scale to clinical assessments may be informative, but after adjusting for routinely assessed clinical prognostic factors the differences in prognosis are unlikely to be of a clinically important magnitude. Future studies might investigate more intensive treatments and more regular clinical reviews to mitigate risks of poor prognosis for those reporting a severe lack of social support.

## 1 Introduction

The majority of adults treated for depression will not remit with the first treatment they receive.^1,2^ There is a lack of evidence to guide clinicians on what information can be gathered pre-treatment to better inform prognosis for depressed patients.^3,4^ Such knowledge can aid the future clinical management of the patient's condition, and many clinicians and patients want to know what the patient's prognosis is.^5^

Studies asking patients about the things they consider to be particularly important to their short-term and longer-term prognoses have highlighted social support as a key factor.^6^ There is no universally accepted definition of social support so for the purposes of this article we propose a working definition as an individual's perception that they are cared for, esteemed, loved, or valued by their peers, friends, or family, and are part of a social network that can be mobilized when needed.^7,8^ Social support is somewhat related to other social factors such as loneliness and social isolation, although they are considered distinct from one and other.^9^ Loneliness is sometimes defined as the gap between desired social contacts (both the amount of them and perceived quality of them) and the social contacts one experiences.^10^ In contrast, social isolation is often defined as the objective rather than subjective rating of the quantity and mobilization of one's social network.^10^ Social support has been found to be an important modifiable target for preventive interventions ^11^ and has been raised as a priority area by clinicians and health policy makers over recent years.^9,12–14^ The association between social support and the onset of depression is well established,^11,15,16^ and hence in this study, we focus on social support alone, without also studying social isolation or loneliness. There are a number of proposed mechanisms by which better social support might help mitigate against depression, for example perceiving oneself as belonging to a supportive network has been associated with a number of positive health outcomes.^7^ In addition, social support can act as a buffer against stress,^17^ whether that be because members of a social network help solve stress-related problems, stop one from directly facing the impact of stressful situations, or by mitigating the impact of stress, this buffering against stress might reduce the probability of depression occurring.^7^ Unlike other known prognostic factors for depression, social support might be modifiable,^11^ and as such, knowing whether it is associated with prognosis could be of clinical value.

Despite evidence for an association with the onset of depression, the association between social support and prognosis is not well evidenced.^18^ Findings from our recent meta-review ^4^ suggest that only four previous systematic reviews have reported on the associations between social support and prognosis for adults with depression.^10,16,19,20^ Those reviews found some limited evidence that low social support is associated with poorer prognosis; however, they contained very few primary studies investigating the effect. There were also a number of methodological problems with the reviews including the combination of different prognostic outcomes (such as treatment response and relapse),^19^ combinations of outcomes at very different post-baseline end points (from two weeks to two years),^19^ and a combination of different ways of measuring and quantifying social support,^10,16,20^ making it difficult to interpret sources of heterogeneity. There was also a lack of clarity on the setting and context of recruitment of participants,^10,20^ and combinations of some treated samples with mainly community-based, non-treated samples,^10^ making it difficult to determine the generalizability of the findings. Most of the primary studies reviewed by the past reviews were cohort studies in which there were typically few people with depression, and most had not sought treatment. As such, inferences about the association between social support and prognosis have been imprecise and might not generalize to the population of treatment-seeking patients seen in clinical practice. Further, none of the reviews investigated the association between social support and prognosis regardless of the type of treatment received (we call this prognosis “independent of treatment”), so the question remains as to whether or not social support is only associated with particular types of treatment, for example, antidepressants as studied in one previous review,^20^ or is associated with prognosis in general. To answer such a question, data are required from participants that received a range of commonly available treatments, delivered to a set standard, so the effects of treatment can be controlled for in a model of prognosis. This ensures that results are generalizable, at least to patients that may receive one of those treatments. In addition, the prior reviews did not investigate the potential for individual items of a measure of social support to capture the association between social support and prognosis. Such findings might have important utility in clinical settings in which completing a full scale measuring social support may not be possible given time pressures. It is also noteworthy that past studies and reviews have either rarely included data from primary care settings, or have not given sufficient information about the settings participants were recruited from to know whether the results are generalizable to other health service settings. In the UK, as elsewhere, the majority of adults with depression initially seek help from primary care settings.^21–23^ Identifying prognostic factors in a primary care sample, independent of treatments, would therefore have important utility.

In a recent study, we found that depressive symptom severity was the strongest indicator of prognosis independent of treatment and we found that a number of other clinical features of depressive illness (depressive “disorder characteristics”) including the durations of depression and comorbid anxiety, comorbid panic disorder and a history of antidepressant treatment, were also independently associated with prognosis.^4^ No prior studies have investigated associations between social support and prognosis independent of these clinical markers of severity, so we do not know whether assessing for social support might prove informative for the clinical management of depression over and above these factors that should be, and often are, routinely assessed in clinic.

### 1.1 Aims of the study

This study aimed to 1) investigate whether social support is associated with prognosis for adults with depression independent of treatment, and independent of depressive “disorder characteristics”; and 2) to investigate whether individual items of a scale measuring social support are associated with prognosis independent of treatment and depressive “disorder characteristics”.

## 2 Methods and Materials

### 2.1 Identification and selection of studies

The protocol for identifying studies and a description of the formation of the Depression in General Practice (Dep-GP) individual patient data (IPD) dataset was pre-registered (PROSPERO: CRD42019129512 (01/04/2019)), details including the process of developing and amending the protocol have been described elsewhere,^3^ and further details are also given in the Supplementary Materials.

We conducted scoping searches to identify the most commonly used diagnostic screening tool and symptom measure used in randomized controlled trials (RCTs) recruiting adults with depression in primary care, that included depressive symptoms and a wide range of anxiety disorders and symptom.This was in order to ensure that studies included here would have data on the range of clinical prognostic factors or depressive “disorder characteristics” that might be routinely assessed in clinic, so that we could ascertain whether or not social support is informative of prognosis in addition to those clinical factors. From those searches, we identified that this was the Revised Clinical Interview Schedule (CIS-R).^24^ The use of this measure at baseline was therefore made an inclusion criterion for our searches. Keeping this consistent minimizes bias in harmonizing data across studies.

In brief, in the final searches studies were identified via searches on bibliographic databases (Medline, Embase, PsycINFO, and Cochrane Central, searched from inception to December 1, 2020), hand-searching of reference lists, and contacting experts for unpublished or missed studies. Search terms included variations of phrases such as “depression” or “major depression”, “RCT” or “Randomized Controlled Trial”, and “CIS-R” or “Clinical Interview Schedule”. See Table S1 for a full list and results of the searches.

A single reviewer (JB) screened titles and abstracts of potentially eligible studies, these were then read in full and judged against inclusion/exclusion criteria by two reviewers (JB and GL) with consultation with a third (SP) to resolve any uncertainties by consensus.

#### 2.1.1 Inclusion and exclusion criteria

Studies were included in the IPD if they were RCTs of adults seeking treatment for depression from a general practitioner/family physician, with unipolar depression confirmed via the revised clinical interview schedule (CIS-R)^24^ at baseline. Studies in the present analyses also had to use the Social Support Scale from the Health and Lifestyle Survey ^25^ at baseline.

Details of the measures used in the included studies from Dep-GP that are relevant to the analyses described here are in Table 1.

#### 2.1.2 Data extraction

The included studies are detailed in Table 2. Data were extracted for each study participant on all variables in Table 3 by the chief investigators or data managers of each individual study. Data were cleaned one study at a time, independently by two reviewers (JB and RS), and cross-checked with publications and via liaison with chief investigators for each study. Issues were resolved by consensus between four reviewers (JB, RS, GL, and SP).

#### 2.1.3 Data integrity checks

Integrity of all baseline and endpoint data for each study was checked with the study team and against publications from each study. The numbers of participants included here are very slightly different than those in the published articles about the individual studies as we removed patients (two from IPCRESS and one from PANDA) with missing data on over 75% of baseline variables.

### 2.2 Ethical considerations

All studies were granted NHS Research ethical approvals and all participants gave informed consent, see Table S2. No additional ethical approval was required for this study: HRA reference 712/86/32/81.

### 2.3 Data analysis plan

#### 2.3.1 Outcomes

Primary Outcome: Depressive symptom scale score at 3–4 months post-baseline. Five studies used the BDI-II at the primary end point, with one using the PHQ-9 only at that point (COBALT, although both the PHQ-9 and BDI-II were used at baseline in that study, Table 2). These outcomes were made comparable between studies in two ways: 1) the standardized mean (“z-score”) of the primary depressive symptom measure score used at 3–4 months post-baseline in each study. The score at 3–4 months was divided by the standard deviation for that measure across all studies, calculated at 3–4 months. 2) The logarithm (“log outcome”) of 3–4 months depression scale scores combined across all studies irrespective of the measure used (this was controlled for by including the random allocation in each study in all models, as detailed below).

Secondary Outcomes: 1) remission on the BDI-II or PHQ-9 at 3–4 months (for definitions see (Table 1)); and 2) z-score of the depressive symptom scale at 6–8 months postbaseline (available in four studies).

#### 2.3.2 Predictors under consideration

Potential baseline predictors of outcome were the total social support score as a continuous variable (sum of all eight items eachscored 1–3) and in three categories defined by the original scale authors: a severe lack of social support (scoring below 19); a moderate lack of social support (scores between 19 and 23); and no lack of social support (scoring 24), these were modeled using dummy variables for severe and moderate lack of social support. Individual items of the Social Support Scale were also investigated as continuous variables. To make estimates across the social support variables comparable, each continuous variable was z-score standardized.

#### 2.3.3 Confounding

Following our protocol ^3^ thetreatment randomization in each study was adjusted for in all models, we then added factors *a priori* considered to be important confounders (age, gender, marital status, and employment status) determined with the use of directed acyclic graphs ^26^ and consideration of associations between the potential confounders and outcomes from previous studies using similar data.^4^ We then adjusted for the BDI-II score at baseline, and then additionally adjusted for the duration of anxiety, the duration of depression, comorbid panic disorder, and a history of treatment with antidepressant medications.

#### 2.3.4 Assessing properties of the social support scale

Before conducting the primary analyses, an exploratory principal component analysis was conducted to identify any distinct underlying components within the Social Support Scale that may inform later analyses. As the eight-item version of the scale had not previously been validated (although a scale containing the first seven items has been^25^, analyses were conducted to determine the internal consistency, split-half reliability, discriminant validity, and latent structure of the Social Support Scale, using an Item Response Theory (IRT) based analysis, see Supplementary Materials (including Table S3) for details, and results from these analyses.

#### 2.3.5 Primary analyses

For each social support variable and each outcome, we built four models sequentially adding confounding variables in the order described above.

Two-stage DerSimonian and Laird random effects meta-analyses were conducted using “admetan” in Stata.^27^ Analyses could have been performed using one-stage approaches as have been conducted in other IPDs.^2,28^ The approaches would have given very similar results here although as the two-stage approach is considered less prone to bias in determining between-study effects it considered the most suitable.^27^

All outcomes were modeled with linear regression, except for remission for which logistic regression were fitted. For the log outcome, exponentiating the coefficient for the prognostic indicator (exposure) variable gives an estimate of the percentage difference in symptoms per unit-change in the exposure variable relative to the mean (ie, this is not a measure of pre-post treatment change in symptoms). It can be expected that the two methods of capturing the primary outcome will give similar results in terms of the direction of association between the social support variables and outcome, but as percentage differences might be more easily understood by patients and clinicians, results when using the log outcome might have greater clinical utility.

The degree of heterogeneity was assessed using prediction intervals and its impact assessed using the *I* ^2^ statistic.^29^

### 2.4 Sensitivity analyses

Sensitivity analyses were conducted where heterogeneity was problematic (eg, with *I* ^2^ above 75%), removing the study contributing most to the heterogeneity, and removing any studies that were rated as having moderate or high risks of bias, or that offered a low quality of evidence. Further sensitivity analyses were conducted using the multidimensional IRT conversion of BDI-II scores and PHQ-9 scores at 3–4 months post-baseline to the PROMIS T-score, and the same analyses using the BDI-II score at 3–4 months in the five studies that had these data. In addition, a quadratic relationship between outcome at 3–4 months and the total score on the Social Support Scale was assessed.

### 2.5 Data handling and data management

Details of the pre-processing stages and handling of missing data including specifications for multiple imputation performed in each study can be found in the study protocol.^4^

### 2.6 Risk of bias and evidence quality

Two reviewers (JB & RS) independently rated the risk of bias in each study using the Quality in Prognosis Studies (QUIPS)^30^ and rated the quality of evidence for each prognostic indicator using the Grading Recommendations, Assessment, Development and Evaluations (GRADE) framework.^31^

## 3 Results

### 3.1 Characteristics of the included studies

**Figure 1:**
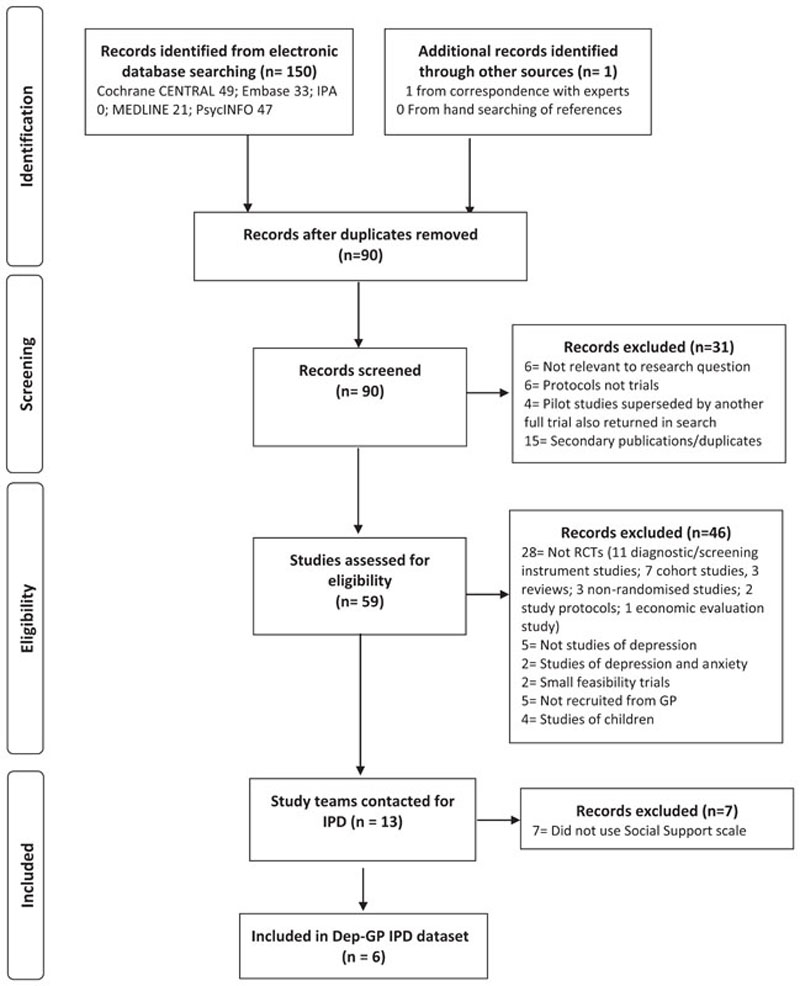
Flow diagram of study selection

In total, six RCTs met the inclusion criteria. IPD from all 2858 participants formed the present dataset, see Figure 1.

### 3.2 Quality assessments and risk of bias

Risk of bias was low and quality was high in all studies, so no sensitivity analyses were required in relation to these, see Table S4. There was near perfect agreement between the reviewers,with interrater reliability (Cohen's Kappa) k = 0.96 for QUIPS and k = 1.00 for GRADE. Disagreements were resolved in consensus meetings with two further reviewers (SP and GL).

### 3.3 Descriptive statistics

Approximately 67% of the participants were female, age at baseline ranged between 18 and 84 years old (mean (SD) = 42.5(14.1) years), 94% were from white ethnic backgrounds, approximately two-thirds had a history of antidepressant treatment, and one third had been depressed for at least one year at the point of their baseline assessments. On average, participants scored in the severe range on the BDI-II and most had a moderate lack of social support, see Table 3.

The correlation between the total social support score and baseline depressive symptom severity (r = −.29) and the correlation between the total social support score and the z-score of depressive symptoms at 3–4 months (r = −.18) were not strong by conventional standards^32^.

### 3.4 The association between social support and prognosis

The total score on the Social Support Scale was associated with the severity of depressive symptoms at 3–4 months, independent of treatment type and additionally adjusted for age, gender, marital status, and employment status, see Table 4. Controlling for depressive symptom severity the magnitude of effect was reduced, and it was very marginally affected further by additionally adjusting for the depressive "disorder characteristic" variables. There was evidence for differences in prognosis by category of social support based on the total score. After adjusting for all variables above, there was evidence that those with a severe lack of social support had higher depressive symptom scale scores at 3–4 months than those with no lack of social support (difference in z-score at 3–4 months: 0.13(95%CI: 0.03 to 0.23)), the difference in prognosis was particularly stark when comparing those with no lack of social support to those with the lowest scores on the scale percentage difference in depressive symptoms at 3–4 months:−22.83% (95% CI:−35.69 to −7.39)), but there was no evidence for a difference between those with a moderate lack of social support compared to no lack of social support (0.04(95% CI: − 0.05 to 0.12):see Table 4). The z-score and log outcomes gave very similar results in terms of the direction and magnitudes of associations between the social support variables and prognosis.

The findings with the secondary outcomes at 3–4 months were similar to those with the primary outcome: For every z-score increase in the total score on the Social Support Scale at baseline, there was an increase in the odds of reaching remission (Table 5). However, the association was some what weaker with the outcome at 6–8 months (Table 5). In sensitivity analyses using the PROMIS T-score, there was a similar pattern of results to the primary outcome. There was also a similar pattern of results when using the BDI-II score at 3–4 months as an outcome in the five studies that had these data. There was little heterogeneity in the effects so no further sensitivity analyses were deemed necessary.In addition, we found no evidence for a quadratic relationship between the total social support scale score and prognosis at 3-4 months (p =.999)

### 3.5 The associations between individualitems of social support and prognosis

There was evidence that all eight of the Social Support Scale items were associated with prognosis independent of treatment age, gender, marital status, and employment status, see Table 4. However, the magnitudes of association were different between the individual scale items, and there was only sufficient evidence that three items were associated with the outcome after additionally adjusting for depressive symptom severity and depressive “disorder characteristics”. These three items were as follows: 1) whether or not one feels accepted for who one is, by family and friends; 2) whether or not one feels cared about by family and friends; and 3) whether or not one feels supported or encouraged by family and friends, see Tables 4–5. There was no clear evidence of an association between any of the individual items and prognosis at 6–8 months post-baseline, see Table 5. Results of sensitivity analyses were very similar to those of the primary analyses (Table 5).

## 4 Discussion

Social support was associated with prognosis independent of treatment type and after adjusting for socio-demographic factors. Prognosis was poorer for those with a severe lack of social support pre-treatment relative to those with no lack of support. The effects were considerably reduced after adjusting for clinical markers of depressive severity, to the point that differences in prognosis may not be of clinically important magnitudes.^33^ Three of the individual items of the Social Support Scale had larger magnitudes of association and were more consistently associated with prognosis across the outcomes than the other five items. These items were “feeling accepted,” “feeling cared about,” and “feeling supported or encouraged” by friends and family. There was little heterogeneity in the effects, supporting the robustness of these findings.

### 4.1 Findings in context

Recent systematic reviews have suggested that patients consider social support to be among the most important factors influencing their mood and outcomes from treatment,^34^ although there has been very limited evidence of an association between social support and prognosis for depressed patients. Despite this, there has been a growing emphasis on social support and related constructs such as loneliness as potential targets for intervention as they are thought to both affect the likelihood of seeking treatment and of engaging in and completing treatment, and hence effect treatment outcomes.^11,13,35^ The present study found an association between social support and prognosis independent of a range of treatments offered to adults seeking treatment for depression in primary care. Adjusting for depressive severity had a large impact on the magnitude of the associations between social support variables and outcomes. In absolute terms, in the five studies that used the BDI-II at 3–4 months, prior to adjusting for the severity factors the BDI-II scores were approximately 5 points higher at 3–4 months for those with a severe lack of social support compared to no lack of social support. After adjusting for the severity and other confounding factors, the difference in scores was only approximately 1.5 points. This is in keeping with our recent study that showed that depressive symptom severity is the largest singleindicator of prognosis for adults with depression in primary care.^4^ Many prior studies have suggested a multidimensional nature to social support.^8^ However, in this study, all items of the Social Support Scale were found to be highly correlated with a single principal component, and the IRT analysis suggested a single latent factor with each item adequately able to be used to discriminate those with different levels of social support.

### 4.2 Strengths and limitations

To our knowledge, this is the first study to use a large individual patient dataset formed from a number of RCTs to consider the associations between social support and prognosis independent of a number of types of treatment. We selected studies that only recruited participants in primary care so findings could be generalizable to large proportions of adults seeking treatment for depression. Data were extracted, cleaned, and checked by multiple reviewers, adding robustness to the methods.^36^ Studies had to use the same measures to determine diagnosis, assess baseline symptoms, social support and confounders so we can have confidence in the ability to use the same measures in clinical practice to inform prognosis. Although this reduced bias in harmonizing data it limited the number of studies meeting inclusion criteria here. Further, although adjustments were made for a number of potential confounders, including clinical, demographic, and socio economic variables, we cannot rule out residual confounding. Further, it is possible that adjusting for baseline depression severity may have led to underestimating or overestimating the effects of social support on prognosis, as baseline severity might have been affected by levels of social support at baseline. This study was not able to address questions of the causal pathway between social support and prognosis, but adjusting for factors routinely assessed in clinic is important to the discovery of clinically informative findings, regardless of the causal pathways. Hence, we adjusted for baseline severity as this should be routinely assessed in clinic.

In addition, the data used here were self-reported and this may have led to additional measurement error; we might expect those with greater baseline depression severity to be least likely to rate their own social support to be high, increasing bias. However, adjustments were made for all depression severity factors associated with the outcomes and social support variables, not just the severity of depressive symptoms, minimizing the potential for such bias here, and potentially increasing utility.^37^ Further, using a standardized outcome is a method that has been criticized previously but the results using the z-score outcome were similar to those with the log outcome and the secondary and sensitivity outcomes, suggesting the use of the standardized outcome metric did not unduly affect the results.

Finally, overall the scores on the Social Support Scale were high;just under half of the sample scored 21 or over and approximately 29% of the sample had the maximum score of 24 on the Social Support Scale. However, scores under the maximum on the measure used here are indicative of a lack of social support so a highly skewed pattern of responses to the questions of the scale were expected.^25,38^

### 4.3 Implications and conclusions

tI is difficult to assess the clinical importance of prognostic factors. One approach is to compare the differences we observed with estimates for the minimal clinically important difference. Previous work has suggested this is about 17.5% for the BDI-II.^33^ By this criterion, only the difference in prognosis for those with the lowest scores on the Social Support Scale compared to those with no lack of social support (the maximum score) would be considered clinically important. So, assessing levels of perceived social support prior to commencing treatment could be informative for the management of depression. However, only a small proportion of patients are likely to have either the lowest score or maximum score on the Social Support Scale (this was applicable to only 30% of our study sample). Associations were of a lower magnitude after adjusting for clinical prognostic factors and so measuring those in clinic, will be more informative than assessing for social support. Future studies might investigate the causal pathways between social support and prognosis, and whether it is beneficial to directly address a lack of social support by augmenting treatment with interventions targeted at increasing social contacts and improving the quality of relationships with family and friends. There are a number of interventions that are thought to have the potential to affect the number or quality of social relationships which might be particularly worth investigating; these include Interpersonal Psychotherapy,^39^ Dynamic Interpersonal Therapy,^40^ and work with Community Navigators.^41^ Further, future studies might investigate the impact of offering more regular reviews and more intensive treatments to those reporting a severe lack of social support, in order to mitigate the risk of poor prognosis.

Given the psychometric properties of the Social Support Scale, it is reasonable to suggest that any of the eight items could be used to capture aspects of social support although if using only one then feeling “accepted by family and friends,” feeling “supported or encouraged by family or friends,” and feeling “cared about by family or friends,” might be most informative if the whole scale is not able to be used.

Other measures related to but separate from social support, particularly those assessing loneliness and social isolation, could be important additions to assessments in future studies an could prove informative as potential targets for interventions whether in or outside of the therapy room.^16,35,42^ Future research should consider the unique contribution of each of these issues to determining prognosis for adults with depression and any interactions between them.

## Supplementary Material

PRISMA IPD Checklist

Supplementary Materials

## Figures and Tables

**Table 1:** Measures used across the studies of the Dep-GP IPD database

Measure	Details	Scores and cutoffs for remission
CIS-R^24^	Consists of 14 symptom subsectionsscored 0–4 covering core features of depression, depressive thoughts (scored 0–5), fatigue, concentration/forgetfulness, and sleep, generalized anxiety, worry, irritability, obsessions, compulsions, health anxiety, somatic concerns, phobic anxiety (split into agoraphobia, social phobia, and specific phobia), and panic. A final section measures general health, impairment, and weight change	The total score ranges from 0 to 57 with a cutoff of >12 used to indicate likely common mental disorder, primary and secondary diagnoses using ICD-10 criteria are given as are binary indictors of diagnosis for all the disorders assessed. Scores of <12 among those that were previously depressed can be used to indicate remission.
Beck Depression Inventory 2^nd^ Edition (BDI-II) ^43^	Consists of 21 items to assess depressive symptoms, each item is scored 0–3.	There is a maximum score obtainable of 63, and a cutoff of ≥10 is used indicate significant symptoms of depression, scores of <10 are therefore used to indicate remission in those that were previously depressed/scored ≥10.
Patient Health Questionnaire 9–item version (PH–Q9) ^44^	This is a depression screening measure, with respondents asked to rate how often they have been bothered by each of the nine symptom items over the preceding two weeks. Each item is scored 0–3	There is a maximum score of 27 with a cutoff of ≥10 is used to indicate “caseness” for depression, a score of 9 or below for those that were previously depressed is therefore considered to indicate remission
Social Support Scale—adapted by authors of RCTs included in this IPD by adding one item to the Health and Lifestyle Survey Social Support Measure.^25^	An 8–item instrument (the first seven of which are from the Health and Lifestyle Survey) assessing the degree to which participants rated the social support of their friends and family in each of the following domains: 1) being accepted for who one is; 2) feeling cared about; 3) feeling loved; 4) feeling important to them; 5) being able to rely on them; 6) feeling well supported and encouraged by them; 7) being made to feel happy by them; and 8) feeling able to talk to them whenever one might like. Items are scored 1–3, with total scores ranging from 8 to 24; higher scores indicate higher levels of perceived social support. The authors of the Health and Lifestyle Survey suggested the maximum score for social support (which was 21 on that scale) indicated “no lack of social support” scores between 18 and 20 indicated a “moderate lack of social support” and scores of 17 or below indicated a “severe lack of social support”	N/A
Life events:adapted by the authors of Adult Psychiatric Morbidity Surveys ^21^ basedon the Social Readjustment Rating Scale ^45^	Participants are asked to say yes/no to whether they the suffered any of nine events within the last six months, for example, a death/bereavement;being physically attacked/injured; or going through a divorce/separation. Each item is scored yes ^1^ or no (0), and the total score is the sum of all the items.	N/A
Alcohol use: the alcohol use disorder identification test primary care version (AUDIT-PC).^46^	Used to assess alcohol misuse, this includes five items scored 0–4. A cutoff of ≥5 indicates hazardous alcohol use that may be harmful to one’s health	N/A

*Note:* All measures apart from the PHQ–9 were used in all six studies; PHQ–9 was used in three studies (COBALT, MIR,and PANDA).

**Table 2:** Description of included studies

			Age in years	Gender	To depressive symptom severity	T0 CISR-Total Score	T0 social support total score			Depressive symptom outcome measure at 3–4 months
Study	*N*	Inclusion criteria	Mean (SD)	%Female	Mean (SD)	Mean (SD)	Mean (SD)	Remission	Interventions	
COBALT^47^	469	Adults 18–75 with treatment resistant depression, scoring ≥14 BDI-II	49.6 (11.7)	72%	BDI-II = 31.8 (10.7)	30.1 (8.9)	20.0 (3.8)	34%	CBT+TAU vs TAU	PHQ–9
GENPOD ^48^	601	Adults 18–74 with depressive episode	38.8 (12.4)	68%	BDI-II = 33.7 (9.7)	30.8 (8.0)	20.0 (3.8)	41%	Citalopram vs Reboxetine	BDI-II & HADS
IPCRESS ^49^	295	Adults scoring ≥14 BDI-II and GP confirmed diagnosis of depression	34.9 (11.6)	68%	BDI-II = 33.2 (8.8)	29.6 (8.7)	20.0 (3.8)	34%	iCBT+TAU vs TAU +waiting list for iCBT	BDI-II
MIR ^50^	480	Adults ≥18 taking SSRIs or SNRIs at adequate dose for≥6 weeks, and scored ≥14 on BDI-II	50.7 (13.2)	69%	BDI-II = 31.1 (9.9)	27.7 (8.3)	20.5 (4.1)	30%	Mirtazapine vs Placebo	BDI-II & PHQ–9
PANDA ^51^	652	Adults presenting with low mood or depression to GP in last 2 years, free of ADM for 8 weeks up to baseline	39.7 (15.0)	59%	BDI-II = 23.9 (10.3)	21.3 (10.1)	20.6 (3.8)	69%	Sertraline vs Placebo	BDI-II & PHQ–9
TREAD ^52^	361	Adults 18–69 who met diagnostic criteria for MDD and scored ≥14 on BDI-II	39.8 (12.6)	66%	BDI-II = 32.1 (9.2)	28.1 (7.8)	20.1 (3.8)	35%	Physical Activity +TAU vs TAU	BDI-II

Abbreviations: ADM, Antidepressant medication; BDI-II, Beck Depression Inventory; HADS, Hospital Anxiety and Depression Scale; iCBT (internet based therapist delivered cognitive behavioral therapy); MDD, Major Depressive Disorder; PHQ-9, Patient Health Questionnaire—nine item version; T0, Baseline; TAU, treatment as usual.

**Table 3:** Baseline characteristics of study sample

Self-reported baseline characteristics	Factor	*N*(%), or Mean (SD)
Social support	Total score	20.25 (3.85)
	Accepted	2.56 (0.60)
	Cared about	2.75 (0.48)
	Supported or Encouraged	2.53 (0.61)
	Made to feel happy	2.42 (0.64)
	Made to feel important	2.46 (0.66)
	Made to feel loved	2.60 (0.58)
	Can rely on others	2.59 (0.61)
	Can talk to others	2.34 (0.71)
Age		42.52(14.12)
Gender	Female	1900 (67%)
	Male	956 (33%)
	Other	0
Ethnicity	White	2698 (94%)
	Non-White	159 (6%)
Employment status	Employed	1639 (57%)
	Not seeking employment	685 (24%)
	Unemployed	532 (19%)
Marital status	Married/cohabiting	1379 (48%)
	Single	911 (32%)
	No longer married	568 (20%)
Number of recent life events		1.35 (1.24)
Past Antidepressant use	No	908 (32%)
	Yes	1950 (68%)
CIS-R durations	Depression	3.42 (1.37)
	Average Anxiety Duration	2.14 (0.99)
Comorbid panic disorder	No	2623 (92%)
	Yes	235 (8%)
Baseline BDI-II score		30.44 (1.53)
3–4 month BDI-II score		16.07 (11.99)
6–8 month BDI-II score		18.64 (13.44)
Remission 3–4 months	No	1363 (58%)
	Yes	1005 (42%)
Attrition at 3–4 months	No	2382 (83%)
	Yes	476 (17%)

**Table 4:** Outcomes at 3–4 months: difference in Z-score of depressive symptoms (“mean difference”) and percentage difference in depressive symptoms (“%”) per unit increase in baseline Social Support indicator

	Adjusted for treatment only				Additionally adjusted for age, gender, marital status, employment status				Additionally adjusted for baseline depressive symptom severity				Additionally adjusted for depressive “disorder characteristics”‡			
Social Support Indicator	Mean difference (95%CI)	*I* ^2^	%(95%CI)	*I* ^2^	Mean difference (95%CI)	*I* ^2^	%(95%CI)	*I* ^2^	Mean difference (95%CI)	*I* ^2^	%(95%CI)	*I* ^2^	Mean difference (95%CI)	*I* ^2^	%(95%CI)	*I* ^2^
Severe lack vs No lack	0.45 (0.31 to 0.60)	49	44.79 (26.97 to 65.11)	59	0.39(0.23 to 0.54)	55	38.18 (20.30 to 58.71)	61	0.13 (0.03 to 0.24)	10	15.44 (4.46 to 27.57)	28	0.13 (0.03 to 0.23)	0	14.64 (4.25 to 26.06)	22
Moderate lack vs No lack	0.19(0.08 to 0.29)	24	21.04 (9.48 to 33.81)	39	0.17(0.08 to 0.26)	9	18.90 (9.23 to 29.44)	19	0.04 (−0.04 to 0.13)	0	8.77 (1.33 to 16.75)	0	0.04(− to 0.12	0	8.30 (0.84 to 16.31)	0
Per one SD increase Total score	−0.18 (–0.22 to –0.14)	1	−12.59(–15.70 to –9.37)	28	−0.15 (−0.20 to −0.11)	25	−10.87(−14.26 to −7.35)	35	−0.05 (−0.09 to −0.01)	0	−4.27(−7.02 to −1.44)	0	−0.04(−0.08 to −0.01)	0	−4.14(−6.91 to −1.29)	0
Accepted	−0.17 (−0.22 to −0.13)	20	−11.98(−15.60 to −8.20)	48	−0.15 (−0.20 to −0.10)	38	−10.75(−14.77 to −6.54)	57	−0.06 (−0.10 to −0.02)	0	−4.96(−8.21 to −1.58)	9	−0.05(−0.09 to −0.01)	0	−4.68(−7.75 to −1.51)	9
Cared about	−0.14 (−0.18 to −0.10)	0	−9.66(−12.10 to −7.15)	0	−0.12 (−0.16 to −0.08)	0	−8.26 (10.75 to −5.70)	0	−0.04(−0.08 to (−0.08 to −0.01)	0	−3.70(−6.28 to −1.06)	0	−0.04 (−0.08 to −0.01)	0	−3.62(−6.21 to −0.97)	0
Made to feel happy	−0.13 (−0.17 to −0.09)	0	−10.02 (−12.58 to −7.38)	0	−0.11(−0.15 to −0.07)	0	−8.33(−10.95 to −5.64)	0	−0.02 (−0.06 to 0.02)	0	−3.38 (−6.05 to −0.64)	0	−0.02(−0.06 to 0.02)	0	−3.46(−6.13 to −0.72)	0
Made to feel important	−0.13 (−0.17 to −0.09)	2	−9.36(−11.96 to −6.67)	0	−0.10 (−0.14 to −0.06)	0	−7.32(−9.99 to −4.59)	0	−0.01 (−0.05 to 0.03)	0	−1.73(−4.49 to 1.40)	0	−0.001(−0.04 to 0.03)	0	−1.61(−4.37 to 1.24)	0
Made to feel loved	−0.15 (−0.19 to −0.11)	2	−9.97(−13.52 to −6.28)	44	−0.13 (−0.16 to −0.09)	0	−7.93(−11.06 to −4.68)	26	−0.04 (−0.07 to 0.00)	0	−2.60(−5.88 to 1.40)	0	−0.003(−0.07 to 0.00)	0	−2.45(−5.82 to 1.05)	28
Can rely on others	−0.14 (−0.18to −0.10)	7	−9.37(−12.45 to−6. 8	27	−0.11 (−0.16 to −0.06)	30	−7.67(−11.12 to −4.08)	39	−0.04 (−0.08 to 0.00)	11	−2.96(−5.65 to −0.21)	0	−003 (−0.07 to −0.00)	0	−2.69 (−5.32 to −0.2)	0
Supported or encouraged	−0.15 (−0.19 to −0.11)	0	−10.34 (−12.95 to 7.65)	0	−0.13 (−0.17 to −0.09)	0	−9.21 (−11.82 to −6.53)	0	−0.05 (−0.09 to −0.01)	0	−4.45 (−7.13 to −1.69)	0	−0.04 (−0.08 to −0.01)	0	−4.15 (−6.86 to −1.35)	0
Can talk to others	−0.14 (−0.19 to −0.08)	46	−9.91(−13.55 to −6.12)	44	−0.12 (−0.17 to −0.06)	50	−8.53(−12.22 to −4.68)	45	−0.04 (−0.07 to 0.00)	8	−3.47 (−6.24 to −0.63)	0	−0.003 (−0.07 to 0.00)	0	−3.36 (−6.04 to −0.62)	0

*Note:* NB: negative numbers represent lower depressive symptom scale scores at 3–4 months vs mean or reference category, positive numbers represent the oppositet

‡ “disorder characteristics” adjusted for are: average anxiety duration, depression duration, panic disorder, and history of treatment with anti depressants. *I^2^* values were calculated separately for each of the outcome variables and are displayed for each analysis.

**Table 5:** Associations of Social Support with secondary outcomes and sensitivity outcomes, adjusted for treatment, age, gender, marital status, employment status, depressive symptom severity, depressive “disorder characteristics,” andand covariates

	Secondary outcomes				Sensitivity analysis			
	Remission at 3–4 months		Depression z-score at 6–8 months		PROMIS T-Score at 3–4 Months		BDI-II Score at 3–4 Months	
Social support indicator	OR(95%CI)	*I* ^2^	Mean difference (95%CI)	*I* ^2^	Mean difference (95%CI)	I^2^	Mean difference (95%CI)	*I* ^2^
Severe lack vs No lack	0.68 (0.52 to 0.88)	0	0.04 (–0.10 to 0.19)	0	1.84 (0.62 to 3.05)	5	1.52 (0.08 to 2.95)	11
Moderate lack vs No lack	0.89 (0.71 to 1.11)	0	0.02 (−0.10 to 0.15)	0	0.88 (−0.15 to 1.90)	4	0.16 (−0.94 to 1.25)	0
Per one SD increase								
Total score	1.15 (1.04 to 1.27)	0	−0.04 (−0.09 to 0.02)	0	−0.60 (−1.05 to -0.15)	0	−0.54 (−1.06 to −0.02)	0
Accepted	1.11 (1.01 to 1.23)	0	−0.04 (−0.10 to 0.01)	0	−0.68(−1.11 to −0.24)	0	−0.70 (−1.22 to −0.18)	0
Cared about	1.14 (1.03 to 1.26)	0	−0.03 (−0.08 to 0.03)	0	−0.56 (−1.00 to −0.13)	0	−0.47 (−0.98 to 0.04)	0
Made to feel happy	1.09 (0.99 to 1.20)	0	−0.02 (−0.08 to 0.03)	0	−0.39 (−0.81 to 0.03)	0	−0.21 (−0.70 to 0.28)	0
Made to feel important	1.09 (0.99 to 1.20)	0	0.00 (−0.05 to 0.06)	0	−0.12 (−0.56 to 0.32)	0	−0.07 (−0.57 to 0.43)	0
Made to feel loved	1.13 (1.03 to 1.24)	0	−0.05 (−0.11 to 0.02)	30	−0.36 (−0.79 to 0.07)	0	−0.58 (−1.09 to −0.08)	0
Can rely on others	1.12 (1.01 to 1.23)	0	−0.03 (−0.09 to 0.02)	0	−0.43 (−1.11 to −0.04)	0	−0.53 (−0.86 to −0.01)	24
Supported or encouraged	1.14 (1.04 to 1.25)	0	−0.04 (−0.09 to 0.02)	0	−0.64 (−1.06 to −0.21)	0	−0.45 (−0.94 to 0.05)	0
Can talk to others	1.08 (0.98 to 1.19)	0	−0.02 (−0.07 to 0.03)	0	−0.44 (−0.86 to −0.02)	0	−0.34 (−0.83 to 0.15)	0

*Note:* All models adjusted for random allocation in each study, depressive symptom severity, average anxiety duration, depression duration, panic disorder, and a history of antidepressant treatment, gender, age, marital status, and employment status.

## Data Availability

Requests for sharing of the IPD used in this study can be made to the corresponding author, any sharing of data will be subject to obtaining appropriate agreements from the chief investigators or data custodians for each individual trial dataset used here.

## Summations

- Social support was associated with prognosis independent of randomized treatment
- A severe lack of social support was associated with considerably worse prognosis
- Social support should be routinely assessed when adults present with depression

## Limitations

- We adjusted for routinely assessed clinical prognostic factors as this tells us about the import of assessing social support in clinic
- But this might have greatly reduced the apparent size of the associations
- Studying the causal pathways between social support and prognosis for adults with depression would be very informative but this study wa unable to address that question
