# Supplementary material for: Is social support pre‐treatment associated with prognosis for adults with depression in primary care?
Source: Acta Psychiatr Scand. 2021 Feb 16;143(5):392–405. doi: 10.1111/acps.13285 (PMC7610633; doi:10.1111/acps.13285)
Supplement: Supplementary file 1 — Table S1‐S4 [file ACPS-143-392-s001.docx]

# Supplementary Materials

## Details of review of systematic reviews and meta-analyses

Searches were run on the Cochrane database of systematic reviews, the Prospero register of systematic reviews, Embase, and Medline. Details of the search terms and results from the searches can be found in Supplementary Table 1. Across the databases 632 articles remained after removing duplicates, 71 of these were somewhat relevant and were read in full, from which 29 were directly relevant as they identified patient characteristics associated with prognosis for adults with depression. Only four of these 29 studies investigated the association between social support and prognosis.

## Details of search terms and search results for RCTs to form IPD dataset

**Supplementary Table 1.** Bibliographic database searches and results

| **Searches** | **Results** |
| --- | --- |
| **Cochrane CENTRAL Trial Register (searched on 1^st^ December 2020)** |  |
| 1. ("Depression" or "MDD" or "Unipolar" or "Depressive"):ti,ab,kw (Word variations have been searched) | 81260 |
| 2. (“RCT” or "controlled trial" or "randomized controlled trial" or "clinical trial"):ti,ab,kw (Word variations have been searched) | 1061566 |
| 3. ("CIS-R" or "Clinical Interview Schedule" or “Revised Clinical Interview Schedule” or “Clinical Interview Schedule Revised”):ti,ab,kw (Word variations have been searched) | 63 |
| 4. #1 and #2 and #3 | **49** |
| **Embase 1947 to 2020 November 30** |  |
| 1. (depression or Depressive disorder or Major depression or Unipolar depression or MDD).mp. | 7288455 |
| 2. exp controlled clinical trial/ or exp "randomized controlled trial (topic)"/ or exp "clinical trial"/ | 1757626 |
| 3. ("Clinical Interview Schedule" or "CIS-R" or "CISR" or "Revised clinical interview schedule" or "clinical interview schedule revised").af. | 857 |
| 4. 1 and 2 and 3 | **33** |
| **International Pharmaceutical Abstracts 1970 to October 2020** |  |
| 1. (depression or Depressive disorder or Major depression or Unipolar depression or MDD).mp. | 10409 |
| 2. (RCT or controlled trial or randomized controlled trial or clinical trial).mp. | 15679 |
| 3. ("Clinical Interview Schedule" or "CIS-R" or "CISR" or "Revised clinical interview schedule" or "clinical interview schedule revised").af. | 3 |
| 4. 1 and 2 and 3 | **0** |
| **Ovid MEDLINE 1946 to December 01 2020** |  |
| 1. exp major depression/ or exp "depression (emotion)"/ | 122064 |
| 2. exp Depressive Disorder, Major/ | 30626 |
| 3. exp Depressive Disorder, Major/ or exp Depressive Disorder/ or exp Depression/ | 219973 |
| 4. 1 or 2 or 3 | 219973 |
| 5. exp controlled clinical trial/ or exp "randomized controlled trial (topic)"/ | 608159 |
| 6. ("Clinical Interview Schedule" or "CIS-R" or "CISR" or "Revised clinical interview schedule" or "clinical interview schedule revised").af. | 621 |
| 7. 4 and 5 and 6 | **21** |
| **PsycINFO 1806 to November Week 4 2020** |  |
| 1. exp major depression/ or exp "depression (emotion)"/ | 158704 |
| 2. (depression or Depressive disorder or Major depression or Unipolar depression or MDD).mp. | 344487 |
| 3. 1 or 2 | 344715 |
| 4. exp "randomized controlled trial (topic)"/ or exp "clinical trial"/ or exp "controlled trial"/ or exp "randomized clinical trial"/ | 12545 |
| 5. (RCT or controlled trial or randomized controlled trial or clinical trial).mp. | 43921 |
| 6. 4 or 5 | 50453 |
| 7. ("Clinical Interview Schedule" or "CIS-R" or "CISR" or "Revised clinical interview schedule" or "clinical interview schedule revised").af. | 1223 |
| 8. 3 and 6 and 7 | **47** |
| **Cochrane CENTRAL Trial Register (searched on 1^st^ December 2020)** |  |
| 1. ("Depression" or "MDD" or "Unipolar" or "Depressive"):ti,ab,kw (Word variations have been searched) | 81260 |
| 2. (“RCT” or "controlled trial" or "randomized controlled trial" or "clinical trial"):ti,ab,kw (Word variations have been searched) | 1061566 |
| 3. ("CIS-R" or "Clinical Interview Schedule" or “Revised Clinical Interview Schedule” or “Clinical Interview Schedule Revised”):ti,ab,kw (Word variations have been searched) | 63 |

## Details of additions, deviations and changes to protocols

We have registered the process of finding studies and the research questions for this study on PROSPERO (CRD42019129512) and produced a protocol paper which was amended twice. Below we explain the amendments made and the process of finding studies and forming the dataset for this study.

We started this project with one of the senior investigators (GL) in possession of individual patient data from two studies for which he was the chief investigator, and a third study that he was in the process of conducting. We ran scoping searches to identify the most commonly used comprehensive measure of depressive and anxiety disorder symptoms and diagnoses in RCTs of depression in primary care, and noted that this was the CIS-R. It is noteworthy that other clinical interviews are commonly used in research studies but in our searches for primary care based RCTs very few used full clinical interviews including the Schedules for Clinical Assessment in Neuropsychiatry (SCAN) (1) or the Structured Clinical Interview for DSM (SCID) (2). It was more common that only the depression module from the SCID was used. This may be because the CIS-R is self-administered and the others are interviewer rated and thus place a larger burden on research staff or clinical staff time to conduct the interviews. We therefore refined our scoping searches to look for studies that used the CIS-R. An alternative approach might have been to set inclusion criteria related to the outcome measures used instead of the baseline measures, this may have been particularly useful were it the case that a wide range of outcome measures were found in scoping searches which could not reasonably be harmonized together. This was not the case in the scoping searches we conducted. That author (GL) was a co-investigator on a number of other trials that used the CIS-R and we made contact with the chief investigators of those studies to ask for in-principle agreement to access IPD from their trials. We then applied for funding for this project. Once funding was in place we registered our project on PROSPERO, at that point we had run two rounds of searches (scoping searches and one set to inform our funding application), and we had obtained IPD data from four studies. We refined our searches by including two other databases and contacting experts for missed studies, this helped us find further studies. We invited the chief investigators from each of those studies to join the project. We began to collect some further IPD from the studies that had agreed to take part. We then wrote up a protocol paper with information of what we would do with those IPD data once the dataset was complete. We ran further searches and found one more study just before initially submitting the protocol paper. It was a protracted process to gain IPD from that study but the idea was that the Dep-GP IPD dataset would be formed from all of those studies we had found. The Protocol paper was peer-reviewed and we amended it post-review to give more details about this process. The protocol was then accepted for publication. It was amended once more when we decided that our choice of an I^2^ threshold for considering problematic heterogeneity was too high, we dropped it from 80% to 75% for all models and to 50% for the final models, in line with recommendations from Cochrane. We ran the final searches for studies meeting our inclusion criteria a few months before submitting this manuscript for publication and found no new studies meeting our criteria.

Our protocol paper provides information about all data we sought to extract from the included studies and all outcomes of interest. For the present study we were particularly interested in social supports and potential confounders of the association between social support and prognosis. We put together some exploratory DAGs to help consider what those confounders might be and limited the data used for this study to those factors (and social support). Future studies using these data will consider the prognostic associations between other factors at baseline and prognosis. Further, for this study we amended our inclusion criteria slightly to exclude studies that did not include a measure of social support at baseline. There was one change to the statistical analysis plan that should be noted: we did not include attrition as an outcome for the present study.

## Ethical Approvals and Trial Registrations details for studies included in Dep-GP IPD dataset

**Supplementary Table 2.** Ethical approval and Trial Registration details of the studies included in the Dep-GP IPD database

| **Study** | **Ethical Approvals** | **Trial Registration details** |
| --- | --- | --- |
| COBALT | Approvals were granted by West Midlands Research Ethics Committee (NRES/07/H1208/60) and research governance approval was obtained from the local Primary Care Trusts/Health Boards | ISRCTN38231611; https://doi.org/10.1186/ISRCTN38231611 |
| GENPOD | Approvals granted by South West Research Ethics Committee (MREC 02/6/076) and research governance approval was granted by Bristol, Manchester and Newcastle Primary Care NHS Trusts. | ISRCTN31345163; https://doi.org/10.1186/ISRCTN31345163 |
| IPCRESS | Approval granted by Royal Free and Hampstead Research Ethics Committee, reference number 05/Q0501/18 | ISRCTN45444578; https://doi.org/10.1186/ISRCTN45444578 |
| MIR | Approvals were granted by South East Wales Research Ethics Committee Panel C (ref: 12/WA/0353); Bristol Clinical Commissioning Group (CCG), and other CCGs provided research governance assurance. | ISRCTN06653773; https://doi.org/10.1186/ISRCTN06653773 |
| PANDA | Ethical approval was granted by Bristol Research Ethics Committee Centre (12/SW/0267). | ISRCTN84544741; https://doi.org/10.1186/ISRCTN84544741 |
| TREAD | Approvals were granted by West Midlands multicentre research ethics committee (MREC 05/MRE07/42), and research governance approval was given by the relevant local National Health Service primary care trusts | ISRCTN16900744; https://doi.org/10.1186/ISRCTN16900744 |

## Additional Details of Methods and Data Analyses

### Missing Data

Missing data were imputed using multiple imputation with chained equations (MICE) in [Stata](https://www.stata.com/) 15.0. This approach uses regression models to impute missing values. A number of imputed datasets (here we used 50) are produced to reflect the uncertainty/variability in the imputation process. Data not reasonably able to be log transformed to meet normality assumptions, were imputed using predictive mean matching (PMM) via a k-nearest neighbours approach as it is considered to be more appropriate for non-normal continuous variables(3), here we used k=10. Linear regression was used for approximately normally distributed continuous variables, logistic regression models for binary variables, and ordinal and multinomial regression models for ordered and unordered categorical variables respectively. All imputation models were built using data on baseline and outcome variables following conventions (4). Only variables with less than 50% missing data were imputed. All imputation models were run to produce 50 imputed datasets. If the primary analysis showed that results differed considerably when studies with systematically missing baseline data were included/excluded from the meta-analytic models, then a separate imputation approach would have been taken, to impute these systematically missing data using multiple imputation with multilevel random effects for study (5).

### Software & Packages

Stata SE 15(6): ipdmetan(7), mvmeta(8), MICE(9), mi impute pmm(10) packages.

### Additional Details on Assessment of Risk of Bias and Study Quality

Risk of bias assessments were conducted using the Quality in Prognosis Studies (QUIPS) tool (11). There are other risk of bias rating systems specific to IPD datasets but they require the included studies to be predictive modelling studies (12) which was not the RCTs included in the present study. Two reviewers (JB and RS) independently rated the risk of bias in each study related to: i) study participation; ii) study attrition; iii) prognostic factor measurement; iv) outcome measurement; v) study confounding; and vi) statistical analysis and reporting. Studies were then given ratings of “high”, “moderate” or “low”, in relation to risk of bias. The quality ratings were conducted in relation to social support as a potential prognostic factor, and each study was also graded as High, Moderate, or Low quality in accordance with the Grading Recommendations, Assessment, Development and Evaluations (GRADE) framework (13).

## Supplementary Results

### Properties of the Social Support Scale

All eight items were highly correlated with one another (r=(0.60 to 0.82), and a single component solution explained approximately 76% of the variance of the Social Support Scale with all items highly correlated with the principal component (Pearson’s ρ= 0.79 to 0.87), see Table 4. In the IRT analysis the measure displayed excellent model fit with a single dimension (M2(12) = 149.6, p < .0001, root mean squared error of approximation (RMSEA)= 0.06, comparative fit index (CFI)= 0.99 and Tucker-Lewis Index (TLI)= 0.99). Individual item loadings on the unidimensional latent variable ranged between 0.78 and 0.92. There was good internal consistency (Empirical reliability = 0.84; Cronbach's α = 0.91; Guttman’s λ6 = 0.91), split-half reliability (Revelle's β = 0.87), and discriminative validity (corrected item-total correlations: 0.68-0.76).

**Supplementary Table 3.** Correlation Matrix of Social Support Items and Principal Component

| **Correlation between items (Pearson’s ρ)** | | | | | | | | | |
| --- | --- | --- | --- | --- | --- | --- | --- | --- | --- |
|  | **Accepted** | **Cared about** | **Made to feel Happy** | **Made to feel Important** | **Made to feel Loved** | **Can rely on others** | **Supported and Encouraged** | **Can talk to others** | **Principal Component** |
| **Accepted** | 1 |  |  |  |  |  |  |  |  |
| **Cared about** | 0.74 | 1 |  |  |  |  |  |  |  |
| **Made to feel Happy** | 0.64 | 0.78 | 1 |  |  |  |  |  |  |
| **Made to feel Important** | 0.69 | 0.82 | 0.79 | 1 |  |  |  |  |  |
| **Made to feel Loved** | 0.69 | 0.77 | 0.69 | 0.76 | 1 |  |  |  |  |
| **Can rely on others** | 0.74 | 0.75 | 0.67 | 0.71 | 0.76 | 1 |  |  |  |
| **Supported or Encouraged** | 0.74 | 0.78 | 0.76 | 0.76 | 0.73 | 0.76 | 1 |  |  |
| **Can talk to others** | 0.66 | 0.70 | 0.71 | 0.70 | 0.60 | 0.69 | 0.75 | 1 |  |
| **Principal Component** | 0.80 | 0.87 | 0.83 | 0.85 | 0.81 | 0.82 | 0.86 | 0.79 | 1 |

**Supplementary Table 4.** Risk of Bias and Quality Ratings for each of the six included studies.

|  | **QUIPS Risk of Bias Ratings** | | | | | | **GRADE Quality Assessment** |
| --- | --- | --- | --- | --- | --- | --- | --- |
| **Study** | **Study Participation** | **Study Attrition** | **Prognostic Factor Measurement** | **Outcome Measurement** | **Study Confounding** | **Statistical Analysis and Reporting** | **As a Prognostic Indicator** |
| COBALT | Low | Low | Low | Moderate | Low | Low | High |
| GENPOD | Low | Low | Low | Low | Low | Low | High |
| IPCRESS | Low | High | Low | Low | Low | Low | High |
| MIR | Low | Moderate | Low | Low | Low | Low | High |
| PANDA | Low | Low | Low | Low | Low | Low | High |
| TREAD | Low | Low | Low | Low | Low | Low | High |
| **Overall** | **Low** | **Low** | **Low** | **Low** | **Low** | **Low** | **High** |

## Additional References

1. Wing JK, Barbor T, Brugha TS et al. SCAN. Arch Gen Psychiatry 1990;**47**:589.

2. Williams J, Gibbon M, First M et al. The structured clinical interview for DSM-III-R (SCID): II. Multisite test-retest reliability. Arch Gen Psychiatry 1992;**49**:630–636.

3. Horton NJ, Lipsitz SR. Multiple Imputation in Practice: Comparison of Software Packages for Regression Models With Missing Variables. Am Stat 2001;**55**:244–254.

4. Royston P, White IR. Multiple Imputation by Chained Equations (MICE): Implementation in Stata. J Stat Softw 2011;**45**:1–20.

5. Resche-Rigon M, White IR, Bartlett JW, Peters SAE, Thompson SG. Multiple imputation for handling systematically missing confounders in meta-analysis of individual participant data. Stat Med 2013;**32**:4890–4905.

6. StataCorp LP. *Stata Base Reference Manual - Release 15*. College Station, Texas: Stata Press, 2017

7. Fisher DJ. Two-stage individual participant data meta-analysis and generalized forest plots. Stata J 2015;**2**:369–396.

8. White IR. Multivariate Random-effects Meta-regression: Updates to Mvmeta. Stata J Promot Commun Stat Stata 2011;**11**:255–270.

9. Royston P. Multiple imputation of missing values: Further update of ice, with an emphasis on categorical variables. Stata J 2009;**9**:466–477.

10. Morris TP, White IR, Royston P. Tuning multiple imputation by predictive mean matching and local residual draws. BMC Med Res Methodol 2014;**14**:75.

11. Hayden JA, Windt DA Van Der, Cartwright JL, Côté P, Bombardier C. Assessing Bias in Studies of Prognostic Factors. Ann Intern Med 2013;**158**:280–286.

12. Moons KGM, de Groot JAH, Bouwmeester W et al. Critical Appraisal and Data Extraction for Systematic Reviews of Prediction Modelling Studies: The CHARMS Checklist. PLoS Med 2014;**11**:e1001744.

13. Guyatt GH, Oxman A, Vist G et al. GRADE an emerging consensus on rating quality of evidence and strength of recommendations. Br Med J 2008;**336**:924–926.
